# Use of Molecular Gut Content Analysis to Decipher the Range of Food Plants of the Invasive Spotted Lanternfly, *Lycorma delicatula*

**DOI:** 10.3390/insects11040215

**Published:** 2020-04-01

**Authors:** Alina Avanesyan, William O. Lamp

**Affiliations:** Department of Entomology, University of Maryland, College Park, MD 20742, USA; lamp@umd.edu

**Keywords:** insect gut content, invasive species, *Lycorma delicatula*, plant DNA barcoding, trophic interactions

## Abstract

Spotted lanternfly, *Lycorma delicatula* (Hemiptera: Fulgoridae), is an introduced highly invasive insect pest in the US that poses a significant risk to forestry and agriculture. Assessing and predicting plant usage of the lanternfly has been challenging, and little is known regarding the lanternfly nymph association with its host plants. In this study, we focused on: (a) providing a protocol for using molecular markers for food plant identification of *L. delicatula*; (b) determining whether the ingested plant DNA corresponds with DNA of the plants from which the lanternfly was collected; and, (c) investigating the spectrum of ingested plants. We utilized gut contents of third and fourth instar nymphs that were collected from multiple plants; we isolated ingested plant DNA and identified consumed plants. We demonstrated that (a) up to 534 bp of the *rbc*L gene from ingested plants can be detected in *L. delicatula* guts, (b) ingested plants in ~93% of the nymphs did not correspond with the plants from which the nymphs were collected, and (c) both introduced and native plants, as well as woody and non-woody plants, were ingested. This information will aid effective the monitoring and management of the lanternfly, as well as predict the lanternfly host plants with range expansion.

## 1. Introduction

Spotted lanternfly, *Lycorma delicatula* (White), is emerging as one of the most aggressive invasive auchenorrynchan pests in in the eastern US: it is extremely polyphagous and it can feed on over 70 host plants, such as apple, plum, cherry, peach, apricot, grape, pine, tree of heaven (preferred tree host), as well as many ornamental plants [1,2,3,4,5,6]. Nymphs and adults cause severe plant damage through the sucking of phloem sap from the vascular bundles of young stems or leaves, as well as producing honeydew and, consequently, creating conditions for sooty mold [1,6,7]. As nymphs mature, their host plant range decreases; and. they have a few preferred host plants at the late nymphal stage and, especially, adult stage [1]. Exploring the mechanisms of such an unusual use of plant hosts during development (i.e., polyphagous behavior of early instars and nearly monophagous behavior of adults; [1,3]) is instrumental in understanding the lanternfly host plant switch, as well as preventing their rapid infestation of fruit crop plants and, particularly, apples and grapes. Furthermore, it is critical to not only determine a possible host plant range that can be attacked at each nymphal stage, but also confirm the utilization of the host plants for feeding as opposed to resting, migration, laying eggs, etc.

The diet composition of *L. delicatula* has been traditionally explored by field surveys (i.e., observation of the lanternfly occurrence on host plants) and laboratory feeding assays [8,9]. This traditional approach for investigating plant-insect associations is not only time-consuming and challenging, due to, for example, species cryptic coloration or nocturnal activity, but it can also result in taxonomic misidentifications, modifying species feeding behavior in captivity, and other subjective errors [10,11]. In contrast, using molecular biology techniques for plant DNA detection in insect gut contents has been among the most accurate ways to confirm host plant utilization [12,13,14]. This approach overcomes issues with the observational approach, as described above, and potentially represents an unbiased method to record realized plant-insect interactions [10,11,15]. Additionally, the molecular approach can provide higher species resolution and detect plants that were not sampled by the traditional field observations of insect co-occurrence on plants [15].

However, the molecular approach has its own technical limitations [11]. This method becomes even more challenging when dealing with sap-feeding insects as a lack of plant tissue in the gut of a piercing-sucking insect that feeds on phloem makes gut contents difficult to discern. Additional challenges with detecting plant DNA from *L. delicatula* gut contents include, but are not limited to: (a) choice of the plant DNA region, which can be successfully amplified; and, (b) lack of information of feeding behavior of *L. delicatula*, such as feeding time, digestion time, frequency and time for feeding breaks, etc. All of these factors potentially affect the amplification of plant DNA from *L. delicatula* gut contents and plant sequence quality needed for plant identification.

Previous studies on sap-feeding insects, other than *L. delicatula*, showed that issues with molecular approaches still can be overcome, and a portion of various regions of the chloroplast DNA can be successfully detected in insect gut contents [16,17,18]. To date, many studies on the *L. deliculata* feeding preferences have used behavioral or observational approaches [2,3,8,9,19], whereas the protocols for the detection of plant DNA in the lanternfly gut contents are not yet available from literature. Meanwhile, more information on *L. delicatula* biology, behavior, and host range is urgently needed for its effective management [9]. The availability of the detailed protocol for molecular gut content analysis of *L. delicatula* would help researchers not only quickly confirm the ingestion of the plants, but also predict the host plants, which will be more likely attacked by *L. delicatula* at each developmental stage.

In this study we focused on the following objectives to address these issues and develop an effective protocol for plant DNA detection in gut contents of *L. delicatula*: (a) to provide an optimized step-by-step protocol for using molecular markers for identification of plants ingested by *L. delicatula*; (b) to determine whether the detected plant DNA in the insect gut contents corresponds with DNA of the plants from which *L. delicatula* was collected; and, (c) to investigate the spectrum of ingested plant species. We also provide a number of methodological recommendations and potential directions for future studies on the host plant use by *L. delicatula*.

For the purpose of this study, we use the term “host plants” to refer to *L. delicatula* feeding plants—i.e., the plants which *L. delicatula* utilizes as a food source (and not for egg-laying, resting, molting, etc.). Therefore, these are the plants that this insect pest can attack and damage due to its feeding. Based on previous studies on successful plant DNA detection within the gut contents of sap-feeders [18], we expected to detect DNA of ingested plants from the gut contents of *L. delicatula*. We also expected that the detected ingested plant DNA might not correspond with DNA of the plants on which the nymphs were collected. This expectation was based on the extreme mobility of *L. delicatula*, both horizontally and vertically, at all nymphal stages: nymphs ascend and descend their feeding plants from May–July as they develop into mature adults in mid-summer [1,3,4]. Finally, we expected to identify the ingested plants of various life forms and both native and introduced in the US based on a wide range of plants that *L. delicatula* attacks in its native and introduced range [5].

To the best of our knowledge, this is the first study on utilizing molecular markers for detecting plant DNA within the gut contents of *L. delicatula*. We specifically focused on using third and fourth nymphal instars as these developmental stages are extremely polyphagous and they can potentially damage a wide range of plant species [1]. Such protocol will be extremely valuable as a tool for providing evidence of the nymphal feeding on certain plants, determining and predicting the range of feeding plants, as well as assessing the approximate time of food consumption, and potentially the required time that the lanternfly nymphs use between switching food plants. Ultimately, this information is critical for developing effective strategies for the monitoring and management of *L. delicatula*.

## 2. Materials and Methods

### 2.1. Insect and Plant Collection

For this study, we utilized third and fourth instar nymphs of *L. delicatula* collected in Berks County, PA in July 2018. The nymphs were collected from multiple plants from four locations (Locations 1 and 2: field sites with multiple plants; Location 3: a trap tree *Acer rubrum*; Location 4: *Ailanthus altissima*, one tree). *Acer rubrum* and *Ailanthus altissima* were chosen as the preferred host plants for *L. delicatula* [8], to further explore whether observing *L. delicatula* on their preferred host plants demonstrates that the insect feeds on them. From all the locations, the nymphs of *L. delicatula* were collected from a total of 17 various plants. Sequence analysis of the collected leaf samples (described further in Section 2.2) showed that the 17 plants on which the nymphs of *L. delicatula* were collected and from which the leaves were sampled belonged to seven species: *Ailanthus altissima*, *Lonicera maackii*, *Vitis acerifolia*, *Celastrus orbiculatus*, *Rhus typhina*, *Acer rubrum*, and *Acer distylum*. (Table 1).

**Figure 1:**
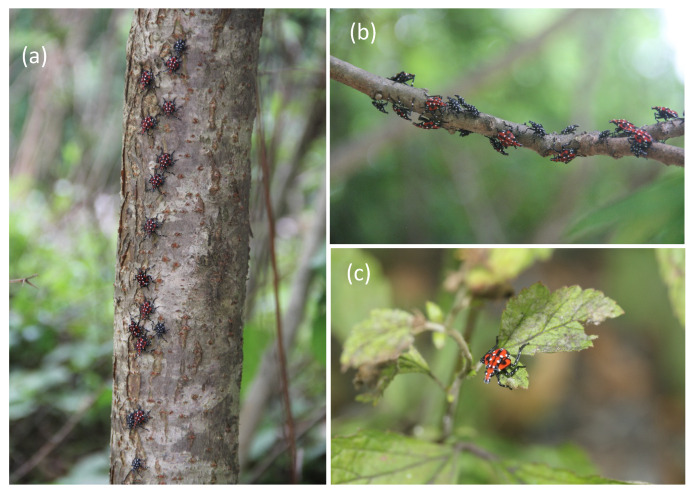
*Lycorma delicatula* third (black) and fourth instar nymphs (red) observed on tree trunks (**a**), tree branches (**b**), and leaves (**c**). (Berks County, PA, USA; July, 2018; Photos by William O. Lamp).

Weather conditions at the collection sites were, as follows: partly cloudy, air temperature ranged from 27–28 °C, and humidity was 39% (retrieved from Weather Underground, www.wunderground.com). The nymphs at the collection sites were constantly moving ascending and descending in the trees; and they were collected from tree trunks, branches, and leaves (Figure 1).

Once collected, the nymphs were immediately preserved in 80% ethanol (to follow the quarantine restrictions and prevent bringing live specimens in Maryland), transported to our laboratory at the University of Maryland, and then stored at +4 °C until the DNA extraction. The DNA extraction was performed within two weeks after insect collection. Following our previous collection procedures [13,20], the leaf samples (hereafter referred as intact plant samples) of each plant the lanternfly nymphs were collected from were immediately dry-frozen, also being transported to the laboratory, and stored at −20 °C until the DNA extraction.

### 2.2. DNA Extraction, PCR, and Sequencing

A total of 41 nymphs of *L. delicatula* (both third and fourth nymphal instars) and 13 intact plant samples (i.e., leaf tissue from 13 different plants from which the nymphs were collected) were selected for DNA extraction. Genomic DNA from both intact plants and individual lanternfly nymphs was extracted while using DNeasy blood and tissue kit (catalog no. 69506, Qiagen Inc., Germantown, MD, USA; www.qiagen.com) following QIAGEN guidelines. Following an approach that was modified from Cooper et al. [18], the body surface of the nymphs was sterilized with 2% bleach solution (by submerging an insect in bleach for 1 min.). The whole insect body and a 10-mm diameter disk from each leaf sample were used for DNA extraction. After DNA isolation, the samples with genomic DNA were either stored at −4 °C or used immediately for PCR amplification.

We modified our previously developed PCR-based method for detecting a non-coding region of plant chloroplast gene (*trn*L intron) from grasshopper gut contents, to provide an optimized step-by-step protocol for using molecular markers for host plant identification of *L. delicatula* [13,20]. In the preliminary testing of the same region of the *trn*L-gene, we were unable to successfully amplify this DNA region from the gut contents of *L. delicatula*: sequences of ingested plant DNA were obtained from <5% of the nymphs (data are not included in this manuscript).

**Figure 2:**
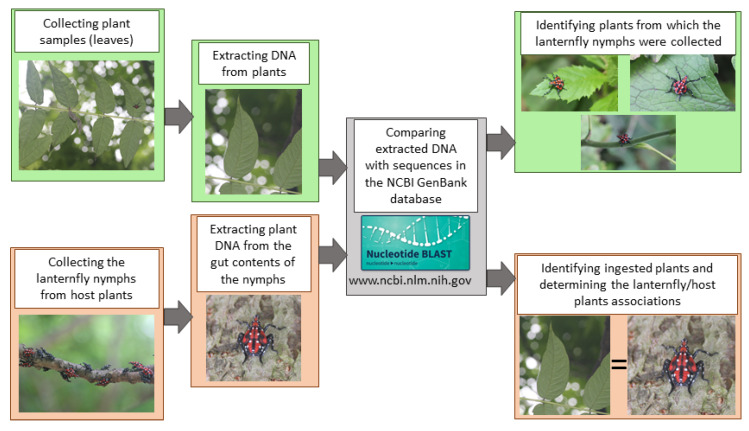
Overview of the main activities of the study: collection *L. delicatula* nymphs and plant samples, DNA isolation from plant samples and *L. delicatula* gut contents, identification plant species, and determining lanternfly-plant associations.

However, for the present study, we utilized a coding region of the chloroplast DNA, *rbc*L gene (codes for ribulose-1,5-biphosphate carboxylase-oxygenase). This region is highly conserved and it is commonly used for the analysis of plant taxonomy and phylogeny, as it is able to resolve higher level relationships across a wide range of plants [21,22]. As the focus of our study was the host plant DNA detection in the lanternfly guts (i.e., developing a tool), rather than precise species identification of ingested plants, the choice of *rbc*L fits our goal best. Primers *rbc*LaF (5′-ATG TCA CCA CAA ACA GAG ACT AAA GC-3′) and *rbc*LaR (5′-GTA AAA TCA AGT CCA CCR CG-3′), which amplify approximately a 530-bp region of the *rbc*L gene, were used to detect plant DNA from both plants and insect gut contents. For each PCR reaction, we used 2μM primers rbcla-F-rbcla-R, 2 μL of each; 10 μL of 2X PCR PreMix, with Dye (Syd Labs Inc., Natick, MA, USA); 5.2 μL of ddH2O; and, 0.8 μL of a template DNA. Thermocycler conditions were set up following manufacturer guidelines for 2X PCR PreMix (the annealing temperature was adjusted for the best amplification results): initial denaturation of 94 °C for 4 min.; followed by 35 cycles of 94 °C for 30 s, 57 °C for 30 s, and 72 °C for 30 s; followed by a final extension of 72 °C for 2 min. 1% agarose gel was used to verify PCR amplification; PCR products were then visualized under a UV transilluminator. PCR products were purified using ExoSAP-IT (catalog no. 78201.1.ML, Affymetrix Inc., Santa Clara, CA, USA), and then sequenced using Sanger sequencing at GENEWIZ (GENEWIZ Inc., South Plainfield, NJ). The sequences were then trimmed and aligned using BioEdit [23], and species identity was determined using BLAST engine in the National Center for Biotechnology Information (NCBI) GenBank database (http://www.ncbi.nlm.nih.gov/genbank/) (Figure 2). The life form and native status (native or introduced) of the ingested plants were retrieved from the USDA PLANT database (https://plants.usda.gov/java/, accessed on 12 March 2020).

### 2.3. Data Analysis

Two values were used to estimate sequence quality: (1) Quality Score (QS) and (2) Contiguous Read Length (CRL). Both values, for each forward and reverse sequences, were obtained from GENEWIZ for each submitted sample (i.e., the gut content from an individual nymph). As indicated by GENEWIZ (genewiz.com), the QS-value represents the average of the quality values (QV) for each base in the sequence [QV = −10log_10_(Pe), where Pe is the probability of error]. The QS-value of 24 and higher indicates a usable sequence with the QS-value of 40 and higher being of very good quality. The CRL-value is the longest uninterrupted length of the sequence that contains bases with the QV-values of 20 and higher.

The QS and CRL values were averaged between forward and reverse sequences for each intact plant sample and insect gut content sample to estimate overall sequence quality, and particularly, the quality of contig sequences which were used for species identification. To compare the quality of both forward and reverse sequences among third and fourth-instar nymphs, one-way ANOVA with a post-hoc TukeyHSD were used. Plant sequences from the collected plant samples were also used as a positive control to evaluate sequence quality from ingested plants. The Shapiro–Wilk and Bartlett tests verified the normality and heteroscedasticity of data, respectively. Data analysis was conducted in R v.3.5.2 [24].

## 3. Results

A total of 41 nymphs of *L. delicatula* (15 third nymphal instars and 26 fourth nymphal instars) were used for host plant DNA isolation. Our sequencing results demonstrated that ingested plant DNA can be reliably detected in *L. delicatula* gut contents. The primers *rbc*La-F and *rbc*La-R successfully amplified a portion of the chloroplast *rbc*L gene from gut contents of 46% of the lanternfly nymphs with the average CRL of 269 ± 27 bp (CRL_max_ = 534 bp). The obtained sequences of the ingested plant DNA showed 84–100% identity with the sequences that are available in the NCBI GenBank database.

Using the *rbc*L gene, we have confidently determined the species identity for the following ingested plants: *Ailanthus altissima*, *Vitis vinifera*, *Celastrus orbiculatus*, *Betula pendula*, and *Acer pseudoplatanus* (Table 2). We have determined the genus for the following ingested plants: *Cucurbita* sp. (possibly *Cucurbita pepo*), *Vitis* sp. (possibly *Vitis rotundifolia*), *Ampelopsis* sp. (possibly *Ampelopsis brevipedunculata*), and *Platanus* sp. (possibly *Platanus occidentalis*). Two ingested plant samples were identified as *Eupatorium compositifolium* and *Rhus typhina* (with ~85% match with available sequences in the NCBI GenBank database); when considering such a low match, it is possible that plants from other genera within the same families (Asteraceae and Anacardiaceae respectively) were ingested. All of the identified ingested plant species are present in Pennsylvania (based on current information available in the USDA PLANT database (https://plants.usda.gov/java/, accessed on 12 March 2020). All of the unique sequences of ingested plant DNA with 100% species match identity are deposited to the NCBI GenBank database (accession numbers: MN856629, MN862495, MT119453, MT108179, and MN862496). Using Sanger sequencing we were not able to obtain the ingested plant DNA sequences from 54% of *L. delicatula* nymphs due to no priming because of low DNA concentration, or mixed DNA templates in the PCR products. Among the successfully sequenced samples, 37% (i.e., seven out of 19 nymphs) were derived from the gut contents from third nymphal instars and 63% (i.e., 12 out of 19 nymphs) of fourth nymphal instars.

**Figure 3:**
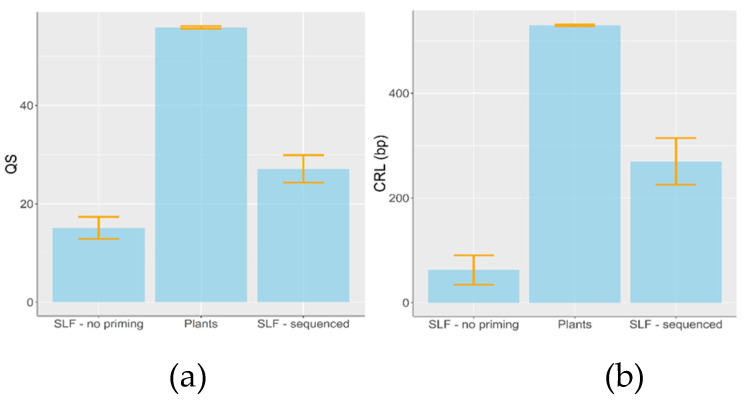
Average sequence quality scores (QS) and contiguous read length (CRL) of the plant sequences isolated from gut contents of *L. delicatula* nymphs (SLF) and from the intact plant samples: (**a**) QS averaged between forward and reverse sequences, (**b**) CRL averaged between forward and reverse sequences. Bars indicate Mean ± SE values.

The average sequence quality scores and contiguous read length did not differ among plant sequences that were isolated from third and fourth nymphs, but they were significantly higher in the sequences isolated from the intact plants (ANOVA; QS_mean_: *F* (2,38) = 11.24, *p* = 0.002; CRL_mean_: *F* (2,38) = 16.32, *p* = 0.0003; Figure 3). Similarly, the QS and CRL of the forward and reverse sequences were significantly higher in intact plants, but did not differ among the nymphal stages (ANOVA; QS: *F* (3,108) = 110.58, *p* < 0.0001; CRL: *F* (3,108) = 53.96, *p* < 0.0001).

**Figure 4:**
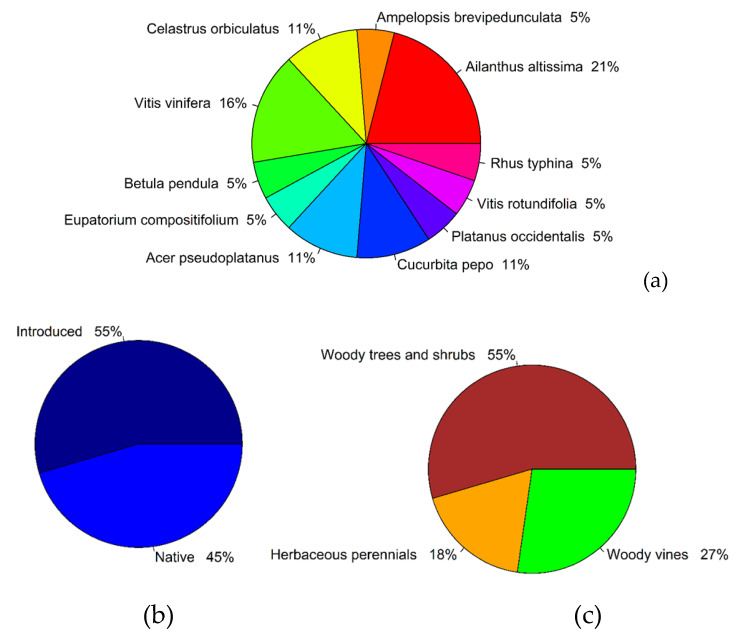
Results of identification of plant species ingested by *L. delicatula* nymphs, based on sequences obtained for a portion of the chloroplast *rbc*L gene: (**a**) Overall proportion of unique plant species isolated from the gut contents of the by *L. delicatula* nymphs (the presented species correspond to the best match in the NCBI GenBank database); (**b**) Proportion of ingested plants with different plant origin (native and introduced in the USA); and, (**c**) Proportion of ingested plants with different life form (woody trees and shrubs, woody vines, and herbaceous plants).

The results of plant DNA identification of intact plant samples showed that the nymphs of *L. delicatula* were collected from seven unique plant species; and, we identified nine unique ingested plant species that were isolated from the lanternfly gut contents (Table 2). Up to seven different plant species were found in the gut contents of the lanternfly nymphs that were collected from one plant species. Only three nymphs, all being collected from *Ailanthus altissima* (samples #12, 18, and 19 in Table 2), showed the DNA in their guts from the plant they were collected from (i.e., *A. altissima*). The rest of the nymphs (~93%) showed the ingested plants other than the plants that they were collected from. We also found that 9% and 27% of nymphs collected from Location 1 (“L1” in Table 2) revealed the ingested plant DNA that corresponds to the plants of the same species and genus, respectively, which were present at the collection site. Among the ingested plants (Figure 4a), 55% of species were introduced and 83% of species were woody plant species (Figure 4b,c). Interestingly, we did detect herbaceous plant species among the ingested plant species (18%) (Figure 4c).

## 4. Discussion

Overall, our results demonstrated that (a) a portion of the chloroplast *rbc*L gene (up to 534 bp) from ingested plants can be reliably detected in *L. delicatula* gut contents, (b) ~93% of *L. delicatula* nymphs, for which the ingested plant DNA was obtained, revealed the ingested plants that did not correspond with the plant species the nymphs were collected from (moreover, the number of ingested plant species was higher than the number of plants on which insects were sampled), and (c) they both introduced and native plant species, as well as woody and non-woody plant species were identified among the ingested plants. We have also detected the ingested plant species that have not been previously reported as the host plants for *L. delicatula* from the eastern US.

### 4.1. Plant DNA Detection from the Gut Contents of L. delicatula

To the best of our knowledge, our study is the first to show that the host plant DNA can be detected in the gut contents of *L. delicatula*. Our findings were consistent with our expectations of the possible detection of ingested plant DNA from a sap-feeder, such as *L. delicatula*: we have successfully isolated a portion of the *rbc*L gene from the most plants consumed by *L. delicatula*: the use of the *rbc*L gene and Sanger sequencing allowed for us to determine the species identity for approximately 55% of ingested plants and the genus identity for 44% of ingested plants.

Previous studies have demonstrated that ingested plant DNA can be obtained from phloem-feeding insects, such as psyllids, due to occasional penetration of the insect stylets through the plant cells [18]. Little is known regarding stylet penetration through the plant tissue by *L. delicatula*, but it is possible that they can also directly rupture phloem cells and, thus, obtain plant DNA in their gut contents. In our recent study on the stylet morphology of *L. delicatula* [25], we found that the tip of mandibular stylets carries additional indentations as the lanternfly grows, which is likely associated with deeper penetration and anchoring in plant tissue while the lanternfly is feeding. It was also shown previously with psyllids that treating insects with bleach does not prevent the plant DNA from being detected suggesting that all of the detected plant DNA is ingested [18]. Our findings similarly showed that all of the detected plant DNA from *L. delicatula* gut contents was ingested.

There are several factors, as discussed below, which could have potentially affected the detectability and identification of host plant DNA within *L. delicatula* gut contents in our study, including (a) the choice of the plant DNA barcode; (b) longevity of ingested plant DNA; and, (c) the presence of a mixed DNA template in PCR products.

The choice of the plant DNA barcode might have affected the number of successfully identified ingested plants at the species level. We used a portion of the *rbc*L gene that is considered to be an excellent plant DNA barcode that is easy to amplify and analyze, and that has a low level of mutation [22]. The *rbc*L gene provides a good resolution at genus level and higher; however, some of the previous studies indicated a lower species resolution of the *rbc*L gene when compared to that of the *trn*L intron [21]. Additionally, not all of the sequences for these DNA regions are available in the NCBI GenBank database, which might prevent the precise species identification using a molecular approach. However, our main focus was to provide evidence that the ingested plant DNA can be detected in the gut contents of *L. delicatula,* as we indicated previously. We did not aim to precisely identify the ingested plant at the species level; nevertheless, we were able to identify ~55% of ingested plants at the species level and ~44% of ingested plants at the genus level; so, we believe this approach can be successfully used for identifying a potential host plant range of *L. delicatula*. Future studies might focus on using a combination of several loci of the chloroplast DNA: it has been suggested in previous studies that using a multi-locus plant barcode (ideally a coding and non-coding region) might provide a higher species resolution and may better resolve phylogenetic relationships [10,21].

Plant DNA longevity in *L. delicatula* gut contents might have affected the successful amplification of ingested DNA from some of the samples that we used. It has been previously suggested in studies with other insects (e.g., the mirid bugs) that DNA from different host plants can show different longevity in insect guts [26]; so, it is possible that some of the plants the lanternfly nymphs consumed before being collected were digested quickly and, therefore, the DNA from those plants was not detected.

Finally, we used Sanger sequencing technology, which allowed us to sequence a DNA fragment from a single plant species only, to obtain sequences of ingested plant DNA. This approach has worked successfully for 46% of the samples (i.e., individual nymphs) that we used in our study. However, through the analysis of sequence chromatogram, we observed that a substantial number of samples (~33%) contained mixed DNA templates. As separating sequences from a mixed DNA template is beyond the capacity of Sanger sequencing, it is possible that some of the ingested plant species could have been missed in our analysis. Therefore, we strongly suggest using metabarcoding of *L. delicatula* gut contents using a next-generation sequencing (NGS) approach for future studies. This can be done targeting the same portion of *rbc*L-gene we used in this study. In fact, in our preliminary testing of this approach using a non-coding region of the chloroplast *trn*L-gene we were able to obtain sequences for >100 ingested plant DNA fragments from the gut contents of one insect individual (third nymphal instar; data are not shown here).

### 4.2. Potential Implications for Host Plant Usage by L. delicatula

In our study, we did not aim to decipher all of the potential host range of *L. delicatula*, but rather we wanted to demonstrate the potential of molecular diet analysis for revealing the consumption of the plants by *L. delicatula* that field observations cannot detect. Thus, our results have confirmed the ingestion of plant tissue from *Ailanthus*, *Acer*, and *Vitis*: heavy lanternfly feeding on these plants was indicated in the previous studies [3,8]. Interestingly, we have detected DNA of *Ailanthus altissima* in the gut contents of all the lanternfly nymphs that were collected from this tree. This also supports the previous findings on the lanternfly feeding preferences for *Ailanthus* [9,27]. We have also found the ingested plant tissue from *Betula* and *Vitis vinifera*: oviposition and feeding on these plants were reported for both China and Pennsylvania [9]. Finally, we have detected (for the first time) the ingestion of *Celastrus* and *Cucurbita*: no previous reports on the consumption of these species by the *L. delicatula* have been published yet.

The presence of a substantial number of introduced plant species in the gut contents of *L. delicatula* is not surprising for such a polyphagous invasive insect. It is also expected that the invasive *L. delicatula* would tend to feed on the plants that are present in their native range, such as *Ailanthus altissima*, which is considered to be its primary host plant [2,27]. However, previous studies demonstrated that other factors in conjunction with the plant origin might also affect the feeding choice of a generalist insect: of those, plant phylogenetic relationships and plant local abundance are especially important [28,29]. Wang et al. [30], for example, showed in their study on the polyphagous mirid bug, *Apolygus lucorum*, that the local composition of host plants at a collection site might greatly affect the data from gut contents of insects that were collected in the field. It would be helpful for future studies to explore a combined effect of these factors and investigate (a) whether native and introduced species that *L. delicatula* feeds on are closely related and (b) whether the most abundant plant species at a field site are most likely to be chosen, regardless of the plant origin or relatedness to other food plants.

Our finding of the presence of non-woody plant species in the gut contents of *L. delicatula* is somewhat surprising: most of the previous studies reported woody plants (trees and vines) as preferred host plants for the lanternfly [2,3,9,27]. Although a few herbaceous species, such as *Metaplexis japonica* (the rough potato), were reported as plants that *L. delicatula* uses to lay eggs in China [9]; to the best of our knowledge, there are no records currently available of using herbaceous plants by *L. delicatula* for egg laying or feeding on the territory of the eastern US. This information is critically important for monitoring *L. delicatula* in its introduced range.

Finally, as we used the Sanger sequencing technology, we detected the ingested plant species that were presumably abundant in *L. delicatula* gut contents and most likely these were the lanternfly’s “last meal”. Given that 36% of the ingested plant samples from site 1 (with the area of ~100 m^2^) “revealed” the species and genus of the plants which were present at this site, it is possible that third and fourth nymphal instars feed on the host plants which grow relatively close to each other and the nymphs do not move far during their meal breaks. Future studies might expand the sample size of *L. delicatula* nymphs and using a larger collection site collect leaf samples from all of the plants that are present at a site; as well as nearby within the insect migration distance; then, similar to our previous study on molecular diet confirmation of grasshoppers [20], further investigations could assess how the distance between plants, plant abundance at a site, and/or plant phylogenetic relatedness affect the lanternfly feeding choice.

### 4.3. Practical Applications, Methodological Recommendations, and Additional Future Directions

The protocol that was developed in our study has many applications for both a better understanding of feeding behavior of *L. delicatula* and in effective management of this pest. Tracking trophic interactions of *L. delicatula* and predicting distribution and movement of *L. delicatula* are among the most important applications.

Specifically, the protocol is a useful tool for tracking the movement and host switch of *L. delicatula* nymphs among various host plants. The molecular gut content analysis is a commonly used approach for tracking trophic interactions [12,20,26,31,32]. For example, in the experiments with the polyphagous mirid bug, *Apolygus lucorum*, while using a DNA-based gut content analysis, it was successfully demonstrated the movement of this pest from cotton to adjacent mungbean fields [26]. It would be particularly helpful for future studies on *L. delicatula* feeding preferences to explore the host plants at each developmental stage and better understand how the host range narrows down at each stage and toward what type of the plants (woody, introduced, etc.). Additionally, as we expected, the majority of ingested plant species that DNA was detected from insect gut contents did not correspond with the tree species on which the nymphs were collected. This evidence supports the fact that the nymphs of *L. delicatula* potentially demonstrate active feeding behavior quickly moving between food plants. This information would be also critical for choosing a trap plant during management of this pest species [17], tracking non-host and shelter plants [33], as well as exploring multiple host plant use [34].

It would be also helpful to include the host plant range, as an additional variable, in modeling the potential distribution of the lanternfly, in addition to climate data, which were previously investigated [7]. Furthermore, it would be interesting to combine this information with the lanternfly morphology. In particular, there is a drastic morphological change in the wing and mouthpart morphology at the adult stage, which might affect *L. delicatula* dispersal and flight tendency [35,36,37], as well as feeding [25]. Additionally, combining DNA barcoding with other techniques, such as stable isotope analysis [38] and/or the immunomarking technique [39], might provide interesting insights into feeding behavior of *L. delicatula*.

From the methodological perspective, feeding behavior and mobility of *L. delicatula* at each developmental stage might have affected the success of the detection of ingested plant DNA in our study. Cadena et al. [11] suggested that large and passive insect herbivores, as well as those which frequently feed may be the best candidate for successful amplification of ingested plant DNA. Therefore, both active late instars that we used in our study, as well as adults that spend most of the time on their preferred host plant [1], should be ideal stages for applying our protocol. We believe that the first and second nymphal instars would not provide successful plant DNA amplification as small active insects might be exposed to various sources of environmental DNA [11].

The chance of successful detection of ingested plant DNA can be also improved by collecting the nymphs (and adults) of *L. delicatula*, while they are actually feeding on a plant rather than moving on the plant surface. This will ensure the present of nondigested plant tissue in the lanternfly guts, which, in turn, will improve the quality of plant DNA template [11,15]. It has also been suggested that insect collection is best done in the morning than afternoon, because of the high foraging activity [15]. Finally, as we mentioned above, supplemental metabarcoding sequencing using a NGS-approach, as well as using multiple chloroplast markers, will improve the identification of the ingested plant fragments that are present at low concentration in the guts of *L. delicatula* [15,21].

## 5. Conclusions

Molecular analysis of the gut contents of sap-feeding insects is a challenging approach due to the quick digestion and/or degradation of plant host DNA. Meanwhile, the availability of the protocol for the DNA-based identification of insect host plants is critical, especially for agriculturally important insect pests. In this study, we demonstrated, for the first time, that the host plant DNA can be detectable from the gut contents of the spotted lanternfly, *Lycorma delicatula*. We have provided a step-by-step protocol for the DNA-based host plant identification of *L. delicatula*, as well as a number of methodological recommendations and potential directions for future studies on host plant use by *L. delicatula*.

## Figures and Tables

**Table 1:** The collection sites description with indication of unique plant species from which *L. delicatula* nymphs were collected (Berks County, PA, USA).

Location	Description	Unique Plant Species ^1^	Nymphs Gut Contents Sequenced ^2^
Location 1	Private territory (backyard); 7 trees; ~ 12 × 8 m^2^ -area	*Lonicera maackii*	Yes
		*Vitis acerifolia*	Yes
		*Celastrus orbiculatus*	Yes
		*Rhus typhina*	Yes
Location 2	Private forest territory; 8 trees; ~ 10 × 10 m^2^ -area	*Ailanthus altissima*	Yes
		*Erechtites hieraciifolius*	No
		*Rubus caesius*	No
		*Acer platanoides*	No
		*Acer distylum*	Yes
		*Phytolacca dioica*	No
		*Eupatorium serotinum*	No
Location 3	Private territory; 1 trap tree	*Acer rubrum*	Yes
Location 4	Private forest territory; randomly chosen tree (preffered host plant)	*Ailanthus altissima*	Yes

^1^ Unique plant species at each location. The plant species identity was determined using the BLAST engine in the National Center for Biotechnology Information (NCBI) GenBank database on 10 March 2020. ^2^ Plant species from which the collected nymphs were successfully sequenced.

**Table 2:** The species identity of plant samples collected in the field and the ingested plants that were obtained from the gut contents of *L. delicatula* nymphs.

#	Instar	Location ^1^	Plants at Field Location ^2^	Ingested Plant			
				Best Match ^3^	Match %	Origin ^4^	Plant Life Form ^4^
1	3	L1	*Lonicera maackii*	*Vitis vinifera*	100	introduced	woody vine
2	3	L1	*Vitis acerifolia*	*Vitis vinifera*	100	introduced	woody vine
3	4	L1	*Vitis acerifolia*	*Celastrus orbiculatus*	98.06	introduced	woody vine
4	3	L1	*Celastrus orbiculatus*	*Ampelopsis brevipedunculata*	94	introduced	shrub
5	4	L1	*Rhus typhina*	*Betula pendula*	100%	introduced	tree
6	4	L1	*Rhus typhina*	*Eupatorium compositifolium*	85.19	native	herbaceous perennial
7	3	L1	*Rhus typhina*	*Vitis vinifera*	100	introduced	woody vine
8	4	L1	*Rhus typhina*	*Acer pseudoplatanus*	100	introduced	tree
9	3	L1	*Rhus typhina*	*Cucurbita pepo*	96.70%	introduced	herbaceous perennial
10	4	L1	*Rhus typhina*	*Acer pseudoplatanus*	99%	introduced	tree
11	4	L1	*Rhus typhina*	*Platanus occidentalis*	95.45%	native	tree
12	4	L2	*Ailanthus altissima*	*Ailanthus altissima*	99%	introduced	tree
13	4	L2	*Acer distylum*	*Rhus typhina*	84%	native	tree
14	3	L3	*Acer rubrum*	*Ailanthus altissima*	96.32%	introduced	tree
15	4	L3	*Acer rubrum*	*Celastrus orbiculatus*	100%	introduced	woody vine
16	4	L3	*Acer rubrum*	*Vitis rotundifolia*	95.22%	native	woody vine
17	3	L3	*Acer rubrum*	*Cucurbita pepo*	85%	introduced	herbaceous perennial
18	4	L4	*Ailanthus altissima*	*Ailanthus altissima*	99%	introduced	tree
19	4	L4	*Ailanthus altissima*	*Ailanthus altissima*	98%	introduced	tree

^1^ See location descriptions in Table 1 (L1: Location 1; L2: Location 2; L3: Location 3; L4: Location 4) ^2^ A tree on which the insect was collected. ^3^ The species identity was determined using the BLAST engine in the National Center for Biotechnology Information (NCBI) GenBank database on 10 March 2020. ^4^ The plant life form and plant origin (i.e., native status in the USA, and particularly in PA) were retrieved from the USDA PLANT database (https://plants.usda.gov/java/, accessed on 12 March 2020).
